# Validation of a Device to Measure Knee Joint Angles for a Dynamic Movement

**DOI:** 10.3390/s20061747

**Published:** 2020-03-21

**Authors:** Mirel Ajdaroski, Ruchika Tadakala, Lorraine Nichols, Amanda Esquivel

**Affiliations:** Department of Mechanical Engineering, College of Engineering and Computer Science, University of Michigan-Dearborn, Dearborn, MI 48128, USA; majdaros@umich.edu (M.A.); rtadakal@umich.edu (R.T.); lvnichol@umich.edu (L.N.)

**Keywords:** internal measurement unit, knee kinematics, knee angles, sports medicine

## Abstract

Participation in sports has risen in the United States over the last few years, increasing the risk of injuries such as tears to the anterior cruciate ligament (ACL) in the knee. Previous studies have shown a correlation between knee kinematics when landing from a jump and this injury. The purpose of this study was to validate the ability of a commercially available inertial measurement units (IMUs) to accurately measure knee joint angles during a dynamic movement. Eight healthy subjects participated in the study. Validation was performed by comparing the angles measured by the wearable device to those obtained through the gold standard motion capture system when landing from a jump. Root mean square, linear regression analysis, and Bland–Altman plots were performed/constructed. The mean difference between the wearable device and the motion capture data was 8.4° (flexion/extension), 4.9° (ab/adduction), and 3.9° (rotation). In addition, the device was more accurate at smaller knee angles. In our study, a commercially available wearable IMU was able to perform fairly well under certain conditions and was less accurate in other conditions.

## 1. Introduction

Over the last few years in the United States, the average participation rate in sports and exercise has risen, from just under 16% in 2003 to 20% in 2015 [1]. While participation in sports is beneficial, there is always the potential for injury. One of the most common types of injuries to occur in many sports, particularly multi-directional sports like soccer and basketball, is injuries to the anterior cruciate ligament (ACL). On average, there are 100,000 to 200,000 ruptures reported every year in the United States alone [2,3]. There are two mechanisms of ACL injury: contact or noncontact. Contact ACL injuries occur as the result of direct contact with another player/equipment and are primarily a singular event. Non-contact ACL injuries can either be acute (a single event) or the result of fatigue due to repetitive, high stress/strain inducing activities. Studies have shown the associated affect fatigue can have on increasing the risk of ACL injury [4]. On average, 70%–84% of all ACL injuries result from this noncontact mechanism, with women being 3–4 times more likely to sustain them [5,6,7,8].

Various studies have noted that movements like cutting, sudden changes in directions, landing from a jump on a single leg, and rapidly stopping all contribute to non-contact ACL injuries [5,9,10,11,12]. Overall, however, it has been observed that a typical non-contact ACL injury occurs when the knee is internally rotated, in slight valgus, and in 10–20° of flexion [13,14]. The theory is, once these conditions are met and a load is applied (e.g., landing from a jump), lateral compression occurs [13,14]. During this lateral compression, and in conjunction with the anterior force caused by muscle contraction (primarily quadriceps contraction), the tibia translates anteriorly with respect to the femur and thereby drastically increases the stress/strain placed on the ACL [15]. The ability to monitor knee kinematics when landing from a jump could allow researchers and clinicians to track instances of these dangerous loading cycles and rest players before a non-contact ACL injury from fatigue occurs.

Traditionally, lower limb joint kinematics have been evaluated under laboratory settings using camera-based motion capture systems [16,17,18]. However, this method cannot be used to track movements in the field. In recent years, wearable sensors have been developed as an alternative to monitoring lower limb joint kinematics, as they could be used outside the laboratory, and have been used in various applications including gait analysis and assistance with rehabilitation [9,11,12,19,20,21,22,23,24,25,26]. Inertial measurement units (IMUs), a particular type of wearable technology, are small devices that can be fitted onto subjects without encumbering their range of motion. These consist of a tri-axial accelerometer and gyroscope and magnetometer. Joint angles of IMUs are calculated as the integral difference between adjacent sensors’ angular velocities, with the initial orientation determined through accelerometer and magnetometer readings. Filtering of these calculated angles is always performed, often utilizing a Kalman filtering process post calculation. However, there are several drawbacks to IMU usage such as artifact motion and consistent placement of the devices. Several studies have validated the accuracy of IMUs to measure knee angles in tasks such as gait analyses and sit-to-stand, and have found moderate to strong correlations between their selected IMUs and a motion camera system [27,28,29,30,31]. Because IMUs have been shown to be accurate in these settings, we wanted to determine whether they could be used to measure knee angles during more dynamic motions. Therefore, the purpose of this study was to validate the joint angles obtained from a commercially available IMU when landing from a jump.

## 2. Materials and Methods

### 2.1. Subjects and Instrumentation

**Figure 1:**
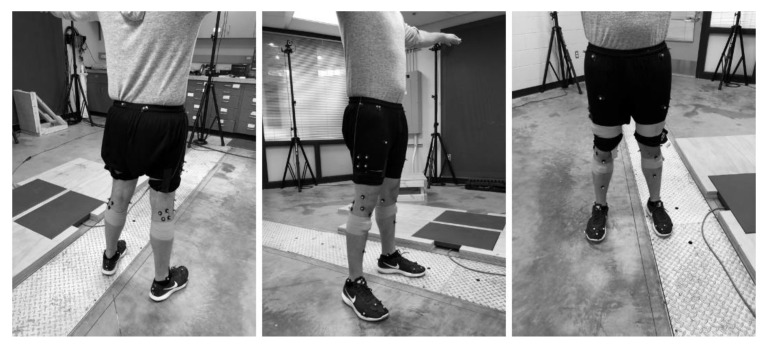
Marker locations.

Eight healthy subjects with an absence of lower extremity injury (five female and three male, 29.0 ± 10.5 years, 76.43 ± 32.43 kg, and 1.71 ± 0.19 m in height) participated in the study (a correlation of R = 0.8 yielded a sample size of 8). All subjects were volunteers and signed an informed consent to participate. The study was approved by the University of Michigan’s (HUM00110145) Institutional Review Board. Subjects were instrumented with motion capture markers located on the right and left lower extremities and wearable motion capture units. A 32 Rizzoli lower body protocol with an additional sixteen retro-reflective spherical markers (25 mm diameter) was placed securely to each subject: (right and left) anterior and posterior superior iliac spine, greater trochanter, thigh, lateral femoral condyle, head of the fibula, anterior tibial tuberosity, shin, lateral malleolus, calcaneus, fifth metatarsal, first metatarsal, distal phalanx, medial femoral epicondyle, medial malleolus, and second metatarsal head (Figure 1). In addition, for the purpose of better defining the constructed virtual model, clusters of four markers were placed on the lateral thigh and gastrocnemius of each leg, resulting in a total of forty-eight markers. Motion and force data were collected using a 12-camera (240Hz) motion capture system (Prime 13 Optitrack, Corvallis, OR) and two 2000 lb force plates (1 kHz) (FP4060-05-PT, Bertec, Columbus, OH).

Four OPAL (APDM Wearable Technologies Inc., Portland, OR) wearable sensors consisting of tri-axial linear accelerometers, gyroscopic, and magnetometers were secured on the medial aspect of the tibia and lateral aspect of the thigh, as recommended by the manufacturer. Data for these sensors were collected at 128 Hz. Orientation of the IMUs was determined from a proprietary fusion algorithm developed by APDM that utilizes accelerometer, gyroscopic, and magnetometer data in conjunction with the application of a Kalman Filter. The method used by APDM is similar to that presented by Watanabe et al. [30]. The relative orientation between the tibia and femur is used to determine the joint angles. The magnetometer provides a reference and orientation is determined via sensor fusion with the linear accelerometers (tilt reference) and gyroscopes (orientation change with time).

A six degree-of-freedom kinematic model of the lower extremity was created for each participant, including the pelvis, thigh, shank, and foot, using Visual 3D software (C-Motion, Germantown, MD). A static trial was collected to determine the participant’s anatomic neutral. Maximum vertical ground reaction force (GRF) as well as maximum resultant GRF were determined from the force plate. Joint angles were calculated in Visual 3D using a cardan rotation sequence (CRS). Joint angles are the relative orientation of one local coordinate system to another and can be represented by three rotations about a unique axis. Emphasis is placed on the order of these rotations. Within this study, a CRS XYZ was implemented, with a lateral rotational matrix determined first, followed by an anterior, and finally a vertical rotational matrix. Additionally, angles obtained through this kinematic model were normalized with respect to the femur through the Visual 3D software.

### 2.2. Testing Procedure

Prior to the start of each trial, subjects were asked to stand with both legs fully extended to allow for initialization of the APDM Opal sensors; this initialization was done so as to eliminate the influence drift may have during calculations. The calibration pose is used to correct for misalignment between both the sensor and segment axes. Subjects were then asked to perform a two-jump action sequence three times. Participants first stood atop a 30 cm wooden box and were asked to jump off onto the force plates. Once on the plates, participants followed through with their initial jump before initiating a second jump before landing again on the plates. While the drop test was uniform across all subjects, the purpose of the second jump was to achieve maximum vertical height, which may differ between the subjects.

### 2.3. Statistical Analysis

Joint angles for motion capture system were recorded at the maximum ground reaction force (mGRF) for each jump and tabulated into excel. For the wearable sensor, joint angles were recorded at the maximum vertical linear acceleration (mVLA). Other studies have found that there is a strong correlation between ground reaction force and linear acceleration [32]. The general relationship between the data from the wearable IMUs and data from the motion capture system was determined through Pearson’s correlation coefficient (R) [33]. Additionally, data were checked to ensure normality and the removal of statistical outliers. A regression analysis (R^2^) between the two systems was calculated, along with the Bland–Altman limits of agreement [34]. The accuracy was evaluated by root mean squared errors (RMSEs). In addition, a paired t-test was performed to determine the significance of any difference between the values of APDM Opal and those of Prime 13 Optitrack. Differences were considered significant if *p* < 0.05. All statistical analyses within the study were performed through the use of Minitab 18 (Minitab Inc., State College, PA).

## 3. Results

### 3.1. Root Mean Square Error (RMSE) and Linear Regression Model

A total of 96 trials (8 subjects; 2 jumps; 2 legs; 3 trials per subjects) were observed between the right and left legs of 8 subjects (5 female, 3 male). No statistical outliers were observed/omitted and data were determined to be normal. There was a moderate relationship between the wearable sensors and traditional motion capture when examining flexion/extension angle at mGRF (Pearson’s R = 0.58). The RMSE for flexion/extension when comparing the two systems was 8.11°. There was a low linear correlation between the two values (R^2^ = 0.34, *p* < 0.01) (Table 1). A negligible relationship between the two systems was observed when examining abduction/adduction angles at mGRF (R = 0.25). The RMSE between the two values was 4.61°. There was a negligible linear relationship between the two systems for abduction/adduction (R^2^ = 0.06, *p* = 0.02) (Table 1). There was a low relationship between the IMUs and the motion capture system when looking at internal/external rotation at mGRF (Pearson’s R = 0.49). The RMSE for internal/external rotation between the two systems was 4.60°. A negligible linear correlation between the values was observed (R^2^ = 0.24, *p* < 0.01) (Table 1).

### 3.2. Bland–Altman Plots

**Figure 2:**
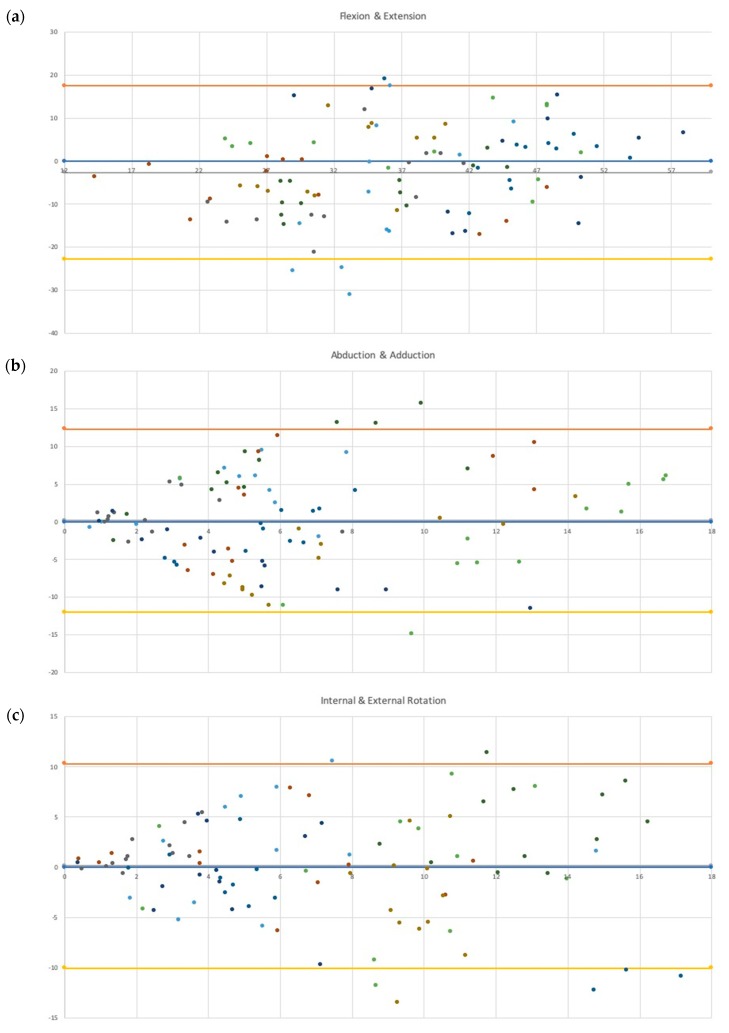
Bland–Altman plot associated with the difference between the (**a**) flexion/extension, (**b**) abduction/adduction, and (**c**) rotation of the inertial measurement unit (IMU) and the motion capture system. The y-axis shows the difference in measured angle between the IMU and motion capture system (Opal-OptiTrack), while the x-axis shows the average measured angle between the two.

The Bland–Altman plot for flexion/extension showed a slight upward trend, indicating that the wearable sensors tend to underestimate angles at lower values and overestimate higher ones. Between 28° and 38°, the highest differences between the systems were observed, with multiple trials falling beyond the bounds of agreement (N = 3 underestimations; N = 1 overestimation) (Figure 2a). The Bland–Altman plot of abduction/adduction showed a tendency for wearable sensors to deviate from the Optitrack camera system as the angle increased. There were nearly equal portions of trials where the wearable sensors overestimated as well as underestimated the value (N = 29 underestimation; N = 26 overestimation). Between 7° and 10°, the highest differences between the systems were recorded, with multiple trials falling beyond the bounds of agreement (N = 1 underestimation; N = 3 overestimation). Beyond 10°, the wearable sensor tended to overestimate the angle (Figure 2b). For internal/external rotation, again, the wearable sensor tended to differ from the camera system as the angle increased. Past 7°, multiple trials were observed to fall outside the bounds of agreement (N = 2 overestimations; N = 5 underestimations), with the greatest concentration occurring at higher angles (past 14° of internal/external rotation) (Figure 2c).

### 3.3. Comparison of Means

There was an absolute mean difference in flexion/extension measurements of 8.43° (95% CI: 7.16° to 9.71°) between the two systems. The observable mean difference was considered significantly different from 0° (*p* = 0.02). For abduction/adduction angles, there was an absolute mean difference of 4.91° (95% CI: 4.17° to 5.66°). The observable mean difference was not significantly different from 0° (*p* = 0.83). The absolute mean difference between the two systems for internal/external rotation at maximum ground reaction force was 3.86° (95% CI: 3.18° to 4.55°). The observable mean difference was not significantly different from 0° (*p* = 0.70).

## 4. Discussion

### 4.1. IMU Comparison

The purpose of this study was to determine whether a set of IMUs could accurately measure knee angle when landing from a jump. This information is useful as it has been shown that the knee may be at risk for injury in this time frame. In this study, we found moderate to negligible linear correlations between the angles obtained through APDM Opal and those obtained through the Optitrack motion capture system. We also observed trends in IMU performance that showed more accurate measurements in abduction/adduction angle and internal/external rotation when the angle was smaller. Flexion/extension examination showed the IMU underestimates angles at smaller values and overestimates larger angles.

Previous studies have compared the accuracy of various IMU sensors to a motion capture system in order to validate them, though they were primarily concerned with knee flexion angles [27,28,30,35,36]. Studies conducted by Tong et al., Takeda et al. and Watanabe et al. all derived angles from the gyroscopic data during a gait analysis [28,29,30]. While the RMSE value observed within our study fell into the range reported by both Tong et al., and Takeda et al. (6° to 9°), it was seen to be much higher than that observed by Watanabe et al.; 8.11° in our study compared with a range between 3° and 4° reported by Watanabe et al. [28,29,30]. Lower RMSE values were seen in studies conducted by both Bakhshi et al. (reported range between 0.08° and 3.06°) and Bell et al. (reported range between 2° and 2.9°), though in both cases, angles were obtained through the IMU software, not the derivation of angular velocity, as was the case in the previously mentioned studies [27,35]. Previous studies have reported considerably higher interclass correlation coefficients with their respective IMUs (range: 0.94 ≤ ρ < 1) than what was observed within our study (0.34) [27,28,29,30]. Though it should be noted that, in all studies, less dynamic actions, such as walking at a normal speed, squatting, and sit-to-stand action, were observed. It is possible that a more dynamic movement, such as a jump, may have caused eccentric gyroscopic fluctuations, which may have influenced angle readings.

The studies conducted by Bell et al. and Zügner et al. also reported the correlation between IMUs and a motion capture system, though they reported intraclass correlation (ICC) [35,36]. While Zügner et al. reported a high ICC between their sensors and motion capture system (ICC > 0.8), Bell et al. reported values that ranged from moderate to high ICC between their two systems (range: 0.58 ≤ ICC ≤ 0.86) [35,36]. The statistical method used to quantify correlation presented by both Zügner et al. and Bell et al., while valid, can be somewhat misleading. Intraclass correlation compares datasets as groups rather than paired observations. Thus, while the intraclass correlation can be high, the interclass correlation can be poor. Of note are the population sizes used in the various studies. While the studies conducted by Watanabe et al. and Bell et al. had population sizes comparable to that of our study (N = 6 and N = 10, respectively), the studies conducted by Bakhshi et al., Takeda et al., and Tong et al. all used much smaller populations (N = 1, N = 3, and N = 2, respectively) [27,28,29,30,35]. The population used by Bakhshi et al. had only a single subject, which may not be enough to establish trends [27].

### 4.2. Potential IMU Performance

Studies have also been performed examining the biomechanics of the knee during actions associated with non-contact ACL injuries. Previous studies examined ACL injuries in women’s handball and basketball, utilizing video sequences analyzed by model-based image matching, medical doctors, and national team coaches [14,15]. It was noted by Koga et al. that all observed ACL injuries fell into one of two categories: cutting actions or one-legged landings [15]. Both groups observed the knee to be fairly straight at the moment of injury, with Koga et al. reporting an average flexion angle of 23° (range of 11° to 30°) and Olsen et al. reporting 16° (range of 5° to 25°) [14,15]. On the basis of our observation of the APDM Opal system performance against the motion capture system, the IMU would have tended to underestimate these flexion angles. This is most readily seen in Figure 1, where the majority of measurements within the ranges reported by the two groups fell below the zero difference line; of 34 trials, 25 fell below this line, with 1 falling beyond the lower limit agreement. For adduction/abduction, Koga et al. and Olsen et al. reported differing observations, with the former reporting the knee to be in a neutral state of 0° (range of –2° to 3°) and the latter reporting an average abduction angle of nearly 14° (range of 5° to 20°) [14,15]. While at lower abduction/adduction angulations, IMU is able to perform fairly accurate, near the upper range of angles reported by Olsen et al., the ability of the IMU to record accurate angles is reduced. For internal/external rotation of the knee, both studies reported the knee to be slightly externally rotated; Koga et al. reported an average external rotation angle of 5° (range of −5° to 12°), while Olsen et al. reported an average external rotation of nearly 2° (range of −15° to 15°) [14,15]. Although, it should be noted that, while reporting an average external rotation angle of 5° at initial contact, Koga et al. observed the knee to internally rotate by 8° (range of 2° to 14°) [14]. The APDM system would be able to accurately record angles at the lower end of this range, though as rotation increases, this accuracy was decreased.

Previously, knee angles have been estimated using video analysis. While video analysis can contribute to the overall understanding of the biomechanics associated with ACL injuries, such as position of the leg, it is limited in that it is not always possible to determine the exact moment of injury [5,37]. This leads to reviewers poorly determining the actual angles of the knee at the moment of injury [5,37]. However, for the purpose of this study, the ranges provided by the video analysis studies provide a basis against which the potential accuracy of angles obtained by APDM Opal could be evaluated. A study conducted by Malinzak et al. compared the knee kinematic patterns between male and female recreational athletes [7]. Across all testing, Malinzak et al. reported that females generally experienced lower flexion angles than their male counterparts, with an approximately 8° difference in angulation between the sexes (*p* < 0.001) [7]. On the basis of the general trends we found, this could mean that this IMU may generally underestimate flexion angles in women and overestimate them in males. Malinzak et al. also observed females consistently experienced higher valgus angles than their male counterparts, with an approximately 11° difference [7]. The IMU measurements for abduction/adduction angles were the least accurate in the 7–10° degree range, indicating that abduction angle measurements recorded in this range may not be accurate [7].

### 4.3. Limitations

We acknowledge that there are several limitations within our study. Wearable sensors cannot be rigidly fixed to the bone and motion artifact may have occurred. However, we attempted to limit this using Co-Flex bands. A study conducted by Allseits et al., using IMUs in gait analysis, also noted the unwarranted affect that noise due to sensor motion may play in proper data analysis [38]. Further, while the effect should not drastically alter any potential findings within our study, further research into the potential effect of sensor motion noise should be done. In addition, our study was not exclusive to athletes. Participants within the study include both athletes and non-athletes. This addition of non-athletes may have swayed the observable ranges for all angles, and thus data ranges may not be indicative of those found in the field. While the marker system recorded data at 200 frames per second, the wearable sensors recorded data at 128 Hz. This difference could have caused some of the discrepancy found between the two systems.

## 5. Conclusions

In our study, APDM IMU wearable sensors were able to perform fairly well under certain conditions and were less accurate in other conditions. Of note is the marked improvement in accuracy when measuring small angular displacement in both abduction/adduction and internal/external rotation, an observation similar to that observed by Taylor et al. [39]. It is possible that these sensors may be able to monitor less dynamic movements more accurately. Determining a better method of securing the IMU to the subject to limit the effect of noise would be helpful. Other studies have investigated drift reduction in highly dynamic motions with other IMUs, indicating a possibility of doing something similar with the sensor used in our study [40].

## Figures and Tables

**Table 1:** Values of the statistical analysis performed on the angles. Mean (SD) and 95% confidence interval (CI) are based on the absolute difference in angle measurement between the inertial measurement unit (IMU) and the motion capture camera. Root mean square error (RMSE) and R-squared (R-sq) value associated with the linear regression model and its significance of fit are also presented.

Statistical Analysis					
	Pearson’s R	RMSE	R-sq	Mean (SD)	95% CI
Flexion/Extension	0.58	8.11	0.34 (*p* < 0.01)	8.43 (6.33)	(7.16, 9.71)
Abduction/Adduction	0.25	4.61	0.06 (*p* = 0.02)	4.91 (3.70)	(4.17, 5.66)
Internal/External Rotation	0.49	4.60	0.24 (*p* < 0.01)	3.86 (3.40)	(3.18, 4.55)
